# Simulation of Carbon Nanotube-Based Enhancement of Cellular Electroporation under Nanosecond Pulsed Electric Fields

**DOI:** 10.1155/2019/9654583

**Published:** 2019-12-13

**Authors:** Yan Mi, Quan Liu, Pan Li, Jin Xu

**Affiliations:** State Key Laboratory of Power Transmission Equipment & System Security and New Technology, Chongqing University, Chongqing 400044, China

## Abstract

Carbon nanotubes (CNTs) with large aspect ratios and excellent electrical properties can enhance the killing effect of nanosecond pulsed electric fields (nsPEFs) on tumor cells, which can improve the electrical safety of nsPEF during tumor treatment. To study the mechanism of the CNT-enhanced killing effect of a nsPEF on tumor cells, a spherical, single-cell, five-layer dielectric model containing randomly distributed CNTs was established using COMSOL and MATLAB, and then, the effects of the addition of CNTs on the electric field and the electroporation effect on the inner and outer membranes were analyzed. The results showed that CNTs can enhance the local electric field strength due to a lightning rod effect, and the closer the CNT tip was to the cell, the greater the electric field strength was around the cell. This increase in the local electric field strength near the cells enhanced the electroporation effects, including pore density, pore area, and pore flux. The simulation results presented in this paper provide theoretical guidance for subsequent development of nsPEF combined with CNTs for use in both cell and tissue experiments.

## 1. Introduction

Nanosecond pulsed electric fields (nsPEF) can induce tumor cell apoptosis and shrink or even cause tumor tissue to disappear, without the participation of toxic chemotherapy drugs, thus alleviating the side effects of inflammation, ulcers and drugs [1, 2], which is of special significance for tumor treatment. However, nsPEF treatments require the introduction of a very high-intensity electric field into tumor tissue by means of electrodes during the experiment. The excessive field strength can easily cause surface discharge of the tumor tissue, damaging the treatment equipment [3, 4], which can lead to electrical safety problems during nsPEF treatments.

In recent years, carbon nanotubes (CNTs) have been widely studied for use in the biomedical field due to their excellent electrical properties. CNTs are one-dimensional quantum materials (their radial dimension is on the nanometer scale, and their axial dimension is on the micron scale) that have high conductivity because the structure of CNTs is the same as that of graphite [5]. CNTs with a large aspect ratio and a high conductivity can enhance the local field strength [6]. Some scholars have applied this property of CNTs to nsPEF treatment of tumors.

Stacey et al. introduced multiwalled carbon nanotubes (MWCNTs) into nsPEF (50 kV/cm, 300 ns, 8 pulses) to kill the pancreatic cancer cell line PANC1 and used trypan blue to evaluate cell viability [7]. Compared with the conditions lacking MWCNTs, the introduction of MWCNTs reduced the cell viability by a factor of 2.3. Due to their unique electrical properties, the MWCNTs were confirmed to act synergistically with the PEF in killing tumor cells. Our group studied the effects of a low-intensity nsPEF combined with MWCNTs on the activity of A375 cells and the rate of apoptosis and necrosis. The results showed that the addition of MWCNTs can simultaneously increase the apoptosis rate and necrosis rate of cells, resulting in a decrease in cell viability [8]. Rojas-Chapana et al. estimated the field strength distribution of the axial positions of CNTs with different aspect ratios by assuming metal strips with flat CNTs [9]. The results showed that the larger the aspect ratio is, the higher the field strength at the axial position is. Through a finite element simulation, Huo et al. found that inserting CNTs with a diameter and length of 10 nm and 1 *μ*m, respectively, into a space with a field strength of 10 kV/m, the field strength at the tip of the CNTs will be distorted to 10^4^ kV/m [10]. From different perspectives, the above studies have verified the feasibility of using CNTs to enhance the killing effect of a PEF on tumor cells.

However, to date, the research on the effect of CNTs in nsPEF treatments has focused on in vitro cell experiments. The mechanism underlying the CNT-enhanced cell killing effect under nsPEF treatment is still unclear, especially the effect of the addition of CNTs on electroporation. The EP model and the Smoluchowski equation are typically used for millisecond to microsecond pulses. However, in recent years, many scholars have employed this model to nanosecond pulse application [11, 12], which provides valuable references to study the mechanism of CNT-enhanced cell electroporation. The killing effect of a nsPEF on tumor cells is inseparable from the electroporation effect on cells. Therefore, to study the mechanism underlying the CNT-enhanced cell killing effect in an nsPEF and provide theoretical guidance for subsequent cell and tissue experiments, this study establishes a dielectric model of CNTs combined with single cells to simulate the effects of CNTs on the spatial electric field distribution and on intracellular and extracellular membrane electroporation.

## 2. Materials and Methods

### 2.1. Geometric Model

**Figure 1:**
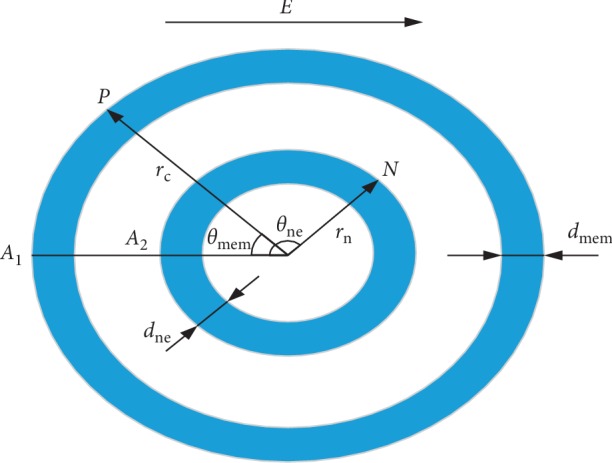
Five-layer dielectric model of a spherical single-cell.

In this paper, a spherical, single-cell, five-layer dielectric model was used for the simulation. A schematic diagram of the model is shown in Figure 1. *E* represents the applied pulsed electric field; *P* and *N* represent arbitrary points on the outer membrane and nuclear membrane, respectively; *A*_1_ and *A*_2_ represent the zero position of the outer membrane and the inner membrane, respectively; *θ*_mem_ and *θ*_ne_ represent the angle between *P* and *N* and the direction of the applied electric field, respectively; *r*_c_ and *r*_n_ represent the radius of the cell and the nucleus, respectively; and *d*_mem_ and *d*_ne_ represent the thickness of the outer cell membrane and the nuclear membrane, respectively.

**Figure 2:**
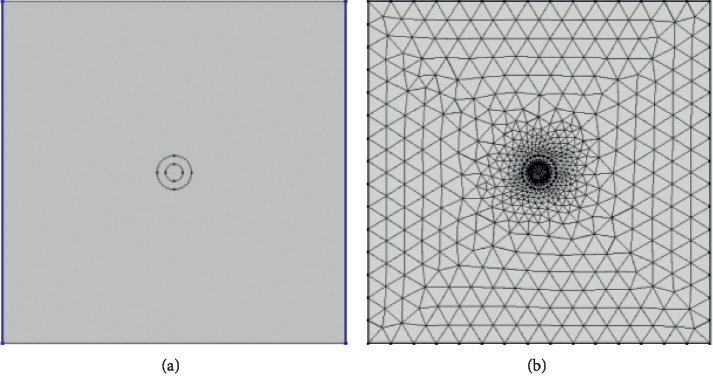
Simulation model without CNTs. (a) Geometric model. (b) Meshing model.

The simulation model and mesh segmentation established with COMSOL finite element software without consideration of CNTs are shown in Figure 2. The entire geometric model is a square with a side length of 200 *μ*m. The PEF is applied to the cells through two metal electrodes positioned opposite each other; the left electrode is connected to the high voltage end, and the right electrode is grounded. The mesh segmentation selects the normal split, and the split model contains 1354 domain units and 108 boundary units. The geometric parameters of the cells are shown in Table 1.

**Figure 3:**
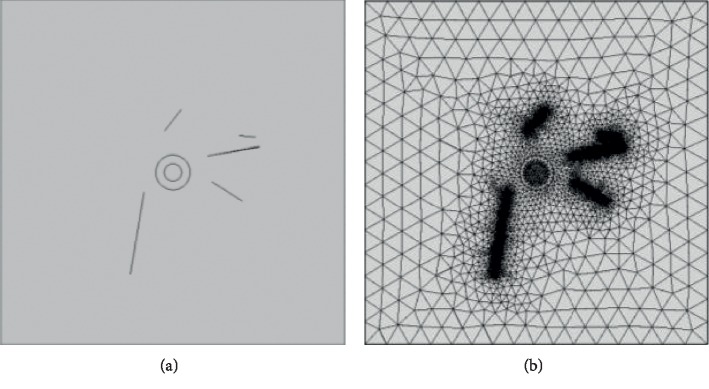
Simulation model with CNTs. (a) Geometric model. (b) Meshing model.

In MATLAB, a script file for COMSOL was created so that COMSOL and MATLAB can access running results generated in the separate packages and both packages can directly use the COMSOL software to complete the simulation of a spherical, single-cell, five-layer dielectric model. Considering the random arrangement of the CNTs around the cells and the agglomeration of the CNTs, their sizes are not necessarily the theoretical values, i.e., the effective length and diameter of the CNTs are random values. In order to more realistically reflect the effect of the introduction of CNTs on cell electroporation, randomly distributed CNTs were added to the spherical, single-cell, five-layer dielectric model using the Monte Carlo algorithm in MATLAB. In previous experiments [8], the density of the CNTs used was 2.1 g/cm^3^, the CNTs were assumed to be cylinders, and the cell concentration was 1 × 10^7^ cells/ml. According to this concentration, the number of CNTs around the single cell was approximately 5. The single-cell simulation model and mesh segmentation of adding multiple, randomly distributed CNTs are shown in Figure 3.

### 2.2. Numerical Model

When the cell is in a region where the applied electric field is *E*, according to the law of the conservation of current in electromagnetic field theory, the potential *ψ* at any point in the space satisfies the following formula:

(1) $$\begin{array}{c} -\nabla \left(\sigma \nabla \psi \right)-\varepsilon_{0}\varepsilon_{\text{r}}\nabla \left(\frac{\partial }{\partial t}\left(\nabla \psi \right)\right)=0, \end{array}$$

where *ε*_0_ is the vacuum dielectric constant and *σ* and *ε*_r_ are the electrical conductivity and relative dielectric constant of the position sought in space, respectively. The transmembrane potential of the cell membrane is described by the following equation:

(2) $$\begin{array}{c} \nabla \psi =\psi_{\text{i}}\left(t\right)-\psi_{\text{o}}\left(t\right), \end{array}$$

where *ψ*_i_ and *ψ*_o_ are the inner and outer membrane potentials of the cell membrane, respectively.

The electroporation of cells under the action of an electric field can be described as the formation of hydrophilic pores on the membrane [13]. The density of the pores on the cell membrane increases, resulting in an increase in the conductivity of the membrane. The micropores can provide a new transport channel for the current. The transmembrane current density flowing through the micropores can be expressed by the increasing term JEP, and the transmembrane current density *J* of the cell membrane can be expressed as follows [14]:

(3) $$\begin{array}{c} J\left(t\right)=\frac{\sigma_{\text{mem0}}\Delta \psi }{d_{\text{mem}}}+\frac{\varepsilon_{0}\varepsilon_{\text{mem}}}{d_{\text{mem}}}\frac{\partial \left(\Delta \psi \right)}{\partial t}+J_{\text{EP}}\left(t\right), \end{array}$$

where the first term in the formula is the ion current density, i.e., the conduction current density. The second term is the capacitance current density, i.e., the displacement current density. The parameter *σ*_mem0_ is the conductivity of the cell membrane when it is not electroporated. According to the transmembrane current density *J*_EP_ expression proposed by Debruin and Krassowska and the Nernst-Planck equation, the variation in the pore density with time can be described by the following Smoluchowski equation [14, 15]:

(4) $$\begin{array}{c} \frac{\text{d}N\left(t\right)}{\text{d}t}=\alpha e^{{\left(\Delta \psi \left(t\right)/U_{\text{EP}}\right)}^{2}}\left(1-\frac{N\left(t\right)}{N_{0}}e^{-q{\left(\Delta \psi \left(t\right)/U_{\text{EP}}\right)}^{2}}\right), \end{array}$$

where *U*_EP_, *q*, and *N*_0_ represent the electroporation characteristic voltage, the effective formation rate coefficient of the electroporation, and the initial pore density, respectively.

The key factor affecting development of the pore size is the energy of the micropores. The detailed process of the micropores from generation and development to disappearance can be obtained through the energy variation law of the pores. For a cell membrane with a total of *K* pores, the pore energy *W* can be obtained from the following equation according to a force model of the micropores on the membrane [16]:

(5) $$\begin{array}{c} W\left(r_{j}\right)=-V_{\text{m}}^{2}F_{\max}\left[r_{j}-r_{h}\ln\left(r_{j}+r_{h}+r_{t}\right)\right]+{\left(\frac{\lambda }{r_{j}}\right)}^{4}+2\pi \gamma r_{j}-\pi \delta_{\text{eff}}r_{j}^{2}, \end{array}$$

where the first to fourth terms of the equation are the energy provided by the electric field force, the spatial repulsive force, the line tension, and the surface tension of the membrane. The parameter *r*_*j*_ is the pore size of the *j*th pore on the membrane, and *j* is from 1 to *K*. The micropore diameter can be obtained by the following differential equation [17]:

(6) $$\begin{array}{c} \frac{\text{d}r_{j}}{\text{d}t}=-\frac{D}{kT}\frac{\text{d}W\left(r_{j}\right)}{\text{d}r}, \end{array}$$

where *D* and *k* are the diffusion coefficients of the pore size and the Boltzmann constant, respectively. Substituting formula (5) into formula (6), an expression describing the aperture changes with time can be obtained [17]:

(7) $$\begin{array}{c} \frac{\text{d}r_{j}}{\text{d}t}=\frac{D}{kT}\left(\frac{V_{\text{m}}^{2}F_{\max}}{1+r_{h}/\left(r_{j}+r_{t}\right)}+4{\left(\frac{\lambda }{r_{j}}\right)}^{4}\frac{1}{r_{j}}-2\pi \gamma +2\pi \delta_{\text{eff}}r_{j}\right). \end{array}$$

The values of the parameters of each physical quantity in the electroporation mathematical model used in this paper are shown in Table 2. The voltage to distance ratio, pulse width of the pulse waveform, and rise time were set to 6 kV/cm, 300 ns, and 2 ns, respectively, and the influence of the CNTs on the space electric field and the electroporation effect of the inner and outer membranes were simulated.

## 3. Results

### 3.1. Spatial Electric Field Distribution

**Figure 4:**
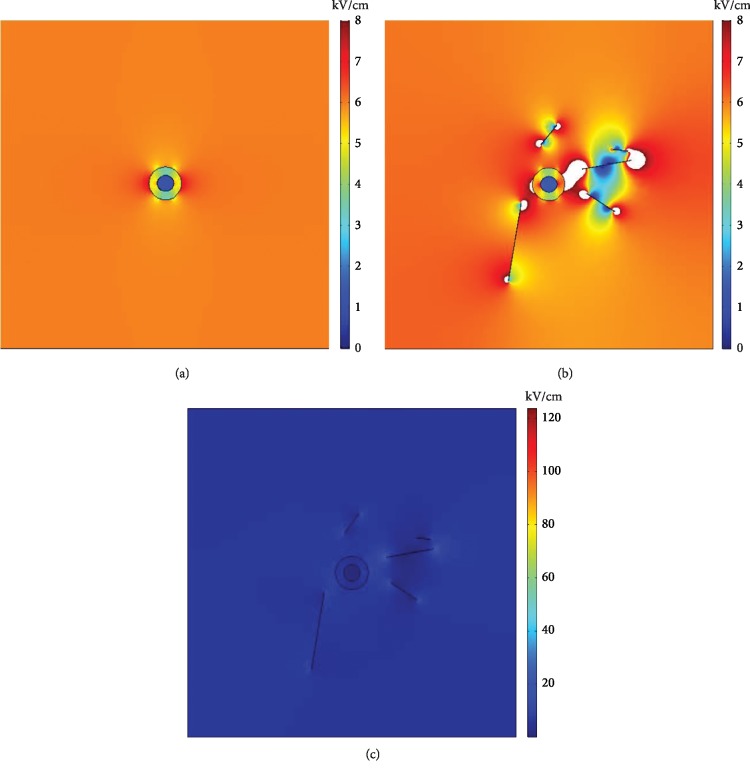
Spatial electric field distribution of the entire plane. (a) Without CNTs. (b) With CNTs (with the same scale shown in Figure 4(a)). (c) With CNTs (original scale).

The electric field distribution results with or without CNTs are shown in Figure 4. Since the electric field span after adding CNTs is too large, to better display and compare the electric field distribution, the scales in Figures 4(a) and 4(b) are set to the same data range (the white sections in *b* represent areas where the electric field strength is greater than 8 kV/cm), i.e., the same color in the two figures represents the same electric field strength magnitude.  Figure 4(c) presents the original scales.

**Figure 5:**
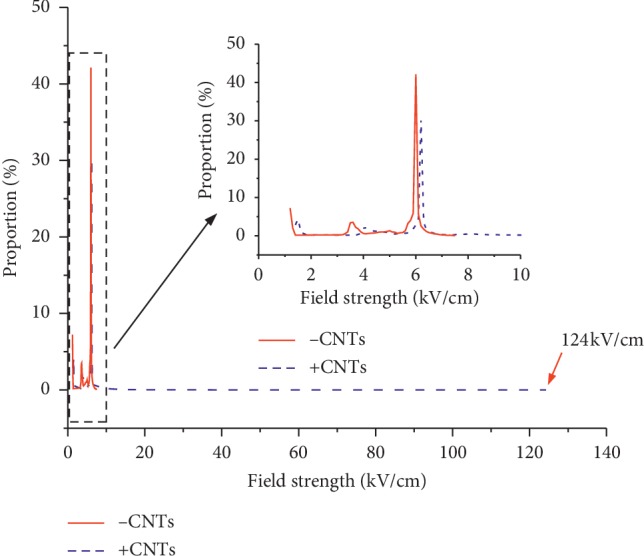
Ratio of the field strength-space distribution area in the entire plane.

The results show that enhancement of the electric field at the tip of the CNTs is particularly obvious. When the applied electric field is 6 kV/cm, the maximum electric field strength in the CNT-free model is located at the left and right poles of the outer membrane, and the maximum electric field strength in the CNT model is at the tip of the CNTs. By integration, the distribution of the area ratio under different field strengths in the entire space can be obtained, as shown in Figure 5.

In Figure 5, the maximum electric field intensity in the space without CNTs can be seen to be 7.5 kV/cm, and the maximum electric field strength in the space when the CNTs are added is 124 kV/cm (Figure 4(c)), which is much larger than the applied electric field.

**Figure 6:**
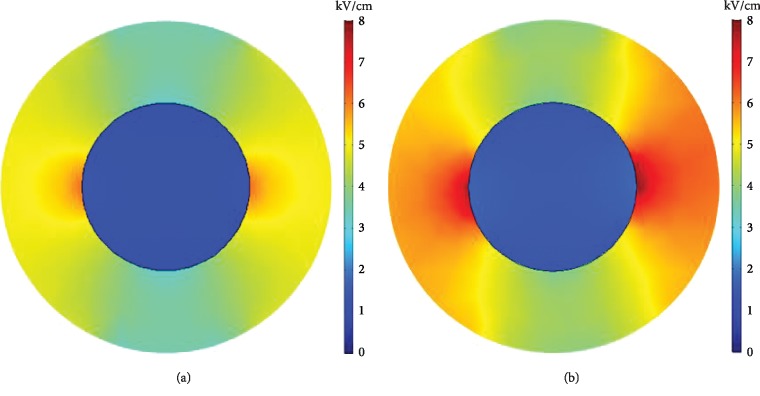
Intracellular spatial electric field distribution. (a) Without CNTs. (b) With CNTs.

**Figure 7:**
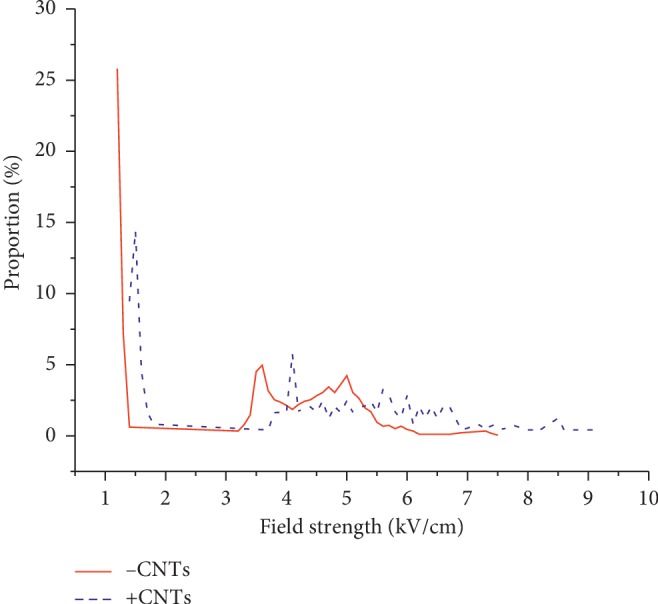
Ratio of the field strength inside the cell to the spatial distribution area.

The electric field distribution in the inner region of the cell and the area distribution ratio under different field strengths are shown in Figures 6 and 7, respectively. The enhancing effect on the internal electric field of the cells after the introduction of CNTs can be more clearly observed. The maximum electric field strength inside the cells was approximately 7 kV/cm without CNTs, and the maximum electric field strength inside the cells when CNTs were added was approximately 9.2 kV/cm.

### 3.2. Electroporation Effect on the Cells

**Figure 8:**
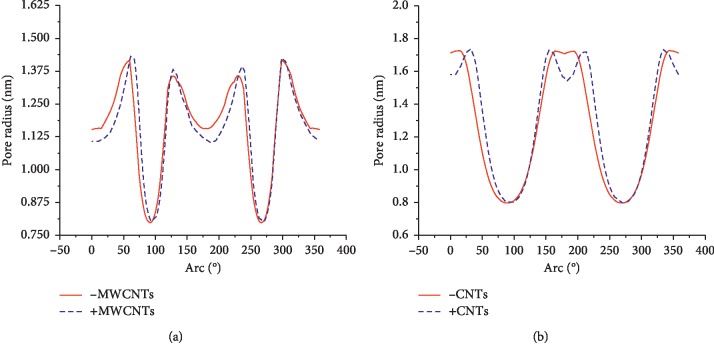
Effect of introduction of CNTs on the pore radius distribution in extracellular and intracellular membranes under a nsPEF. (a) Outer membrane. (b) Inner membrane.

**Figure 9:**
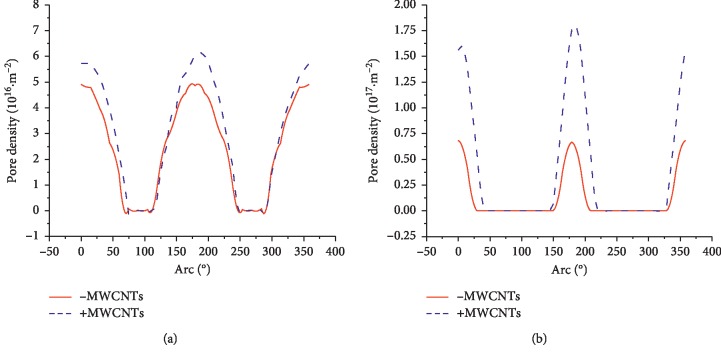
Effect of the introduction of CNTs on the pore density distribution in extracellular and intracellular membranes under a nsPEF. (a) Outer membrane. (b) Inner membrane.

Figures 8 and 9 show the pore size and pore density distribution results for the inner and outer membranes of the cells with and without CNTs, respectively. According to the results, the pore density on the inner and outer membranes of the cells increased after the addition of CNTs. However, the pore diameter on the membrane was not increased at some positions.

Based on the pore size and the pore density, the change law of the membrane electroporation area with time can be obtained as follows:

(8) $$\begin{array}{c} S=\oint \pi r_{j}^{2}N, \end{array}$$

where *r*_*j*_ and *N* represent the pore diameter and pore density, respectively, and the integral path is the circumference along the cell membrane.

**Figure 10:**
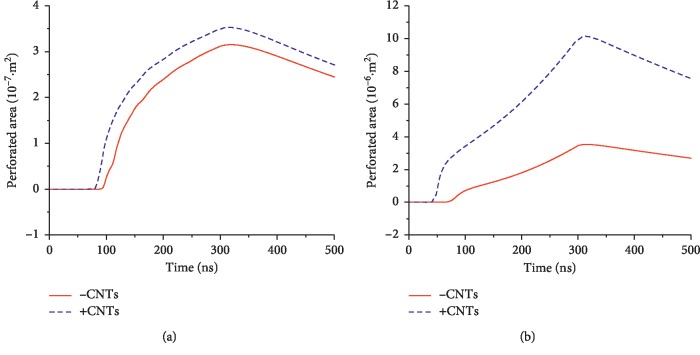
Effect of the introduction of CNTs on the electroporation area of extracellular and intracellular membranes under a nsPEF. The nsPEF was applied at 0 s. (a) Outer membrane. (b) Inner membrane.

Figure 10 shows how the law of the electroporation area of the inner and outer membranes changes with time. The electroporation area of the inner and outer membranes of the cell is close to zero before nsPEF treatment. When electroporation occurs, the electroporation area begins to increase gradually until the pulses are removed. After removal of the pulses, the area begins to decrease due to recovery of the micropores.

**Figure 11:**
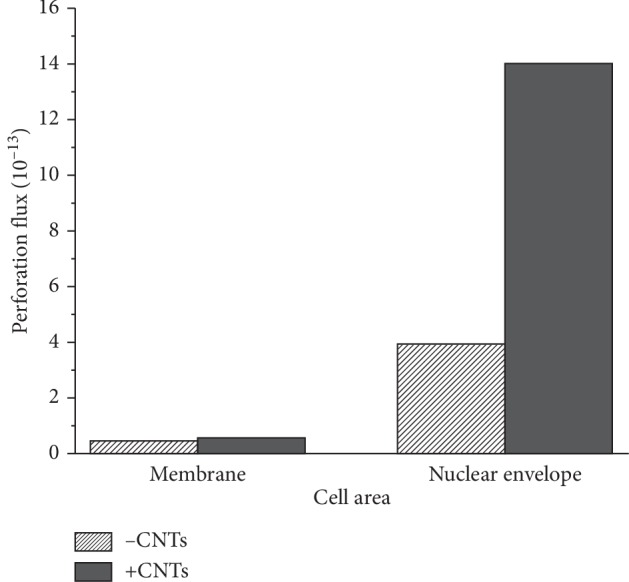
Effect of the introduction of CNTs on the permeabilized flux of extracellular and intracellular membranes under a nsPEF.

The electroporation area of the inner and outer membranes of cells can indirectly reflect how much molecular mass enters and exits the inner and outer membranes of a cell at a certain moment. The cumulative effect of molecular transport can be expressed as the integral of the permeabilized area versus time, defined as the permeabilized flux, which can indirectly describe the cumulative molecular mass of the inner and outer membranes entering and exiting the cell over a period of time. The electroporation area of the inner and outer membranes during the pulse action was time-integrated, and the results are shown in Figure 11. Comparing Figures 10 and 11, it can be seen that the addition of CNTs can significantly increase the electroporation area and electroporation flux of the inner and outer membranes; that is, the CNTs can enhance the electroporation effect on the inner and outer membranes of the cells.

## 4. Discussion

CNTs are one-dimensional quantum materials with special structures (nanoscale in the radial dimension, micron scale in the axial dimension, and almost all ends of the CNTs are sealed). CNTs have unique electrical properties and exhibit good metallicity (a conductivity of approximately 10 S·cm^−1^) [4]. A previous study showed that their current-carrying capacity is 1000 times higher than that of copper wires, making them a three-dimensional conductive matrix in the vicinity of cancer cells [26].

A single CNT with a one-dimensional structure can be simplified into a semispherical metal cylinder placed in a uniform electric field with an amplitude of E_0_. According to the lightning rod effect, the smaller the radius of curvature is, the higher the charge distribution density is. The corresponding local electric field is stronger.

According to the field emission model [27], the electric field enhancement effect at the tip of CNTs can be estimated by the following equation:

(9) $$\begin{array}{c} \frac{E_{\text{tip}}}{E_{0}}=\beta \frac{L}{D}, \end{array}$$

where *E*_tip_ is the electric field strength at the tip of the CNT, *β* is a constant, and *L* and *D* represent the length and outer diameter of CNTs, respectively. The high aspect ratio (*L*/*D*) of CNTs explains why CNTs can effectively concentrate the field and promote electroporation.

For a spherical cell, the electric field intensity of electroporation is described by the following equation [14]:

(10) $$\begin{array}{c} \Delta \varphi =1.5\alpha E\;\cos\;\theta , \end{array}$$

where Δ*φ* is the electroporation transmembrane voltage, *α* is the diameter of the spherical cell, and *θ* is the angle between the normal direction of the target position of the cell membrane and the direction of the electric field (*E*).

When the transmembrane voltage reaches a certain value, electroporation will occur on the membrane. In equation (10), the electric field strength is a key factor that can influence the electroporation effect on a particular cell. In Figures 4 and 5, when the applied field strength is 6 kV/cm, the maximum electric field strength in the space is 7.5 kV/cm without CNTs, and the maximum electric field strength in the space with CNTs added is 124 kV/cm, which is much larger than the applied electric field. The position of the maximum electric field strength is at the tip of the CNTs.

In summary, the addition of CNTs can increase the local field strength of the space, which can further increase the pore density of the permeabilized area on the inner and outer membranes and enlarge the area where electroporation occurs. According to the formula for the development of the pore density, the pore density has an effective relationship with the establishment speed of the microporous transmembrane potential. The higher the field strength is, the faster established the pore membrane potential and the higher the pore density.

Compared with the trend that the pore density increases with increasing field strength, the development of the pore size does not show a similar trend. Even in the electroporation region, the pore diameter decreases with increasing field strength. This behavior occurs because the transmembrane potential of the micropores can move from the rising phase to the falling phase more quickly under the pulse of the high field strength. According to the formula for pore diameter development, the size of the pore depends on the transmembrane potential of the micropore. The higher the field strength is, the earlier saturated the transmembrane potential of the permeabilized membrane in the descending phase. Additionally, a shorter duration of the high transmembrane potential is required to maintain the pore size increase, which means that the pore size is smaller.

In this paper, the electroporation area of the inner and outer membranes and the permeabilized flux were obtained based on the pore size and pore density to comprehensively evaluate the effect of the addition of CNTs on the intensity of intracellular and extracellular membrane electroporation. After the addition of CNTs, the electroporation area and electroporation flux of the inner and outer membranes can be significantly increased; that is, the electroporation effect on the inner and outer membranes of the cells is enhanced. This enhancement of the electroporation effect may make it easier to induce cell apoptosis and necrosis, resulting in an increase in the apoptosis rate and necrosis rate, which leads to a decrease in cell viability.

## 5. Conclusions

In this paper, using COMSOL and MATLAB, a dynamic electroporation mathematical model was applied to establish a spherical, single-cell, five-layer dielectric model containing multiple, randomly distributed CNTs. The effects of the addition of CNTs on the spatial electric field and on intracellular and extracellular membrane electroporation were simulated. The results show that CNTs with large aspect ratios and high conductivity can enhance the local electric field in space. The increase in the local electric field can enhance the electroporation effect on the inner and outer membranes of cells, which is more likely to improve the killing effect of a nsPEF on tumor cells and is of great significance for improving electrical safety during clinical treatments.

## Figures and Tables

**Table 1:** Geometric parameter values of a cell.

Symbol	*r*_c_ (*μ*m)	*r*_n_ (*μ*m)	*d*_mem_ (nm)	*d*_ne_ (nm)
Value	10	5	5	40

**Table 2:** Model parameters for the simulation.

Parameter	Definition	Value
*σ*_m_ (S·m^−1^)	Extracellular medium conductivity [8]	1
*σ*_c_ (S·m^−1^)	Cytoplasmic conductivity [18]	0.3
*σ*_np_ (S·m^−1^)	Nuclear conductivity of cells [19]	1.35
*σ*_mem_ (S·m^−1^)	Cell membrane conductivity [20]	3 × 10^−7^
*σ*_ne_ (S·m^−1^)	Nuclear membrane conductivity [21]	6 × 10^−3^
*σ*_p_ (S·m^−1^)	Pore conductivity [21]	0.22
*σ*_CNTs_ (S·m^−1^)	Carbon nanotube conductivity [8]	1 × 10^8^
*ε*_m_	Relative dielectric constant of the extracellular medium [8]	80
*ε*_c_	Cytoplasmic relative permittivity [22]	154.4
*ε*_np_	Nuclear relative dielectric constant [19]	52
*ε*_mem_	Cell membrane relative dielectric constant [23]	8.57
*ε*_ne_	Nuclear membrane relative dielectric constant [19]	28
*ε*_CNTs_	Carbon nanotube relative dielectric constant [8]	10000
*Ε*_0_ (m^−3^ kg^−1^·s^−4^ A^2^)	Vacuum dielectric constant	8.85 × 10^−12^
*Α* (m^−2^·s^−1^)	Creation rate coefficient [24]	1.0 × 10^9^
*U*_rest_ (mV)	Resting potential [14]	−80
*U*_EP_ (mV)	Characteristic voltage of electroporation [24]	170
*r*_p_ (m)	Minimum radius of the hydrophilic pores [14]	0.8 × 10^−9^
*N*_0_ (m^−2^)	Initial pore density [14]	1.5 × 10^9^
*N*	Relative density of pores [24]	0.15
Ω_0_	Energy barrier coefficient of the pore [24]	2.65
*Q*	Pore creation rate [14]	2.46
*r*_*h*_ (m)	Advection velocity constant [25]	0.97 × 10^−9^
*F* (C·mol^−1^)	Faraday constant	9.65 × 10^−4^
*δ*′ (J·m^−2^)	Lipid-water interface tension coefficient [25]	2 × 10^−2^
*Δ*_0_ (J·m^−2^)	Lipid tension coefficient without electroporation [25]	1 × 10^−6^
*F*_max_ (N·V^−2^)	Maximum electric field force at *V*_m_ = 1 V [25]	0.7 × 10^−9^
*γ* (J·m^−1^)	Lipid-pore energy coefficient [25]	1.8 × 10^−11^
*D* (m^−2^·s^−1^)	Aperture diffusion coefficient [25]	5 × 10^−14^
*R* (J·K^−1^ mol^−1^)	Gas constant	8314
*T* (K)	Absolute temperature	295
*K* (J·K^−1^)	Boltzmann constant	1.38 × 10^−23^

## Data Availability

The data used to support the findings of this study are included within the article.
